# Topics of Nicotine-Related Discussions on Twitter: Infoveillance Study

**DOI:** 10.2196/25579

**Published:** 2021-06-07

**Authors:** Jon-Patrick Allem, Allison Dormanesh, Anuja Majmundar, Jennifer B Unger, Matthew G Kirkpatrick, Akshat Choube, Aneesh Aithal, Emilio Ferrara, Tess Boley Cruz

**Affiliations:** 1 Department of Preventive Medicine Keck School of Medicine University of Southern California Los Angeles, CA United States; 2 American Cancer Society Washington, DC United States; 3 Department of Computer Science University of Southern California Los Angeles, CA United States

**Keywords:** nicotine, electronic cigarettes, Twitter, social media, social bots, cessation

## Abstract

**Background:**

Cultural trends in the United States, the nicotine consumer marketplace, and tobacco policies are changing.

**Objective:**

The goal of this study was to identify and describe nicotine-related topics of conversation authored by the public and social bots on Twitter, including any misinformation or misconceptions that health education campaigns could potentially correct.

**Methods:**

Twitter posts containing the term “nicotine” were obtained from September 30, 2018 to October 1, 2019. Methods were used to distinguish between posts from social bots and nonbots. Text classifiers were used to identify topics in posts (n=300,360).

**Results:**

Prevalent topics of posts included vaping, smoking, addiction, withdrawal, nicotine health risks, and quit nicotine, with mentions of going “cold turkey” and needing help in quitting. Cessation was a common topic, with mentions of quitting and stopping smoking. Social bots discussed unsubstantiated health claims including how hypnotherapy, acupuncture, magnets worn on the ears, and time spent in the sauna can help in smoking cessation.

**Conclusions:**

Health education efforts are needed to correct unsubstantiated health claims on Twitter and ultimately direct individuals who want to quit smoking to evidence-based cessation strategies. Future interventions could be designed to follow these topics of discussions on Twitter and engage with members of the public about evidence-based cessation methods in near real time when people are contemplating cessation.

## Introduction

While combustible tobacco product use is declining in the United States, electronic cigarette (e-cigarette) use has risen in recent years among youth and young adults [1]. Nicotine is the primary psychoactive substance responsible for the abuse potential (ie, the likelihood that a substance will cause addiction) of combustible tobacco products and many e-cigarettes [2]. Like several other psychoactive drugs, including caffeine and amphetamines, nicotine produces acute central nervous system effects including increased heart rate, blood pressure, alertness, and decreased appetite [3], and both animal and human studies suggest that the drug may produce long-term deleterious effects on cognitive development among youth [3,4].

Research has repeatedly shown that there is substantial misunderstanding regarding the health risks of nicotine use [5]. While nicotine is the psychoactive component that sustains tobacco dependence [6], the primary carcinogenic harms are due to combustion of the tobacco leaf [3]. Nevertheless, one study demonstrated that 54% of smokers incorrectly believed that reductions in nicotine made cigarettes less dangerous [7]. Additionally, young adults (a priority population for tobacco control) commonly have misperceptions about the safety profile and nicotine content in e-cigarettes [8], including the unsubstantiated belief that e-cigarettes are relatively safe despite the burgeoning evidence indicating the products’ nicotine-related abuse potential [9,10] and associations with progression to regular combustible cigarette use [11].

Availability of different e-cigarette products like those compatible with multiple substances (eg, open-system pod mods) [12-14] or products that facilitate customization may contribute to youth experimentation and transitions to combustible cigarette use. Such nicotine-use trajectories among youth make it crucial to characterize the public’s experiences with, and perceptions of, nicotine.

Publicly accessible data from people who post to social media platforms, like Twitter, can be used to describe perceptions of nicotine and the social and environment context surrounding nicotine use [15]. Twitter is used by 22% of US adults (distributed fairly evenly through racial and gender groups), with 42% of users on the platform daily [16]. Twitter is also used by 32% of adolescents (13 to 17 years old) in the United States [17]. Previous analyses of posts to Twitter have provided insight about what the public organically discusses regarding tobacco, including the frequency of use, co-use with other substances (eg, alcohol, marijuana), mentions of tobacco product appeal, and the locations where tobacco is often used [18,19]. Past literature also highlights the role of social bots (ie, automated accounts created to produce content and interact with human accounts on Twitter) in spreading unsubstantiated health claims and misinformation on health-related topics such as vaping and vaccines [20,21]. The goal of this study was to identify and describe nicotine-related topics of conversation authored by the public and social bots on Twitter, including any misinformation or misconceptions that health education campaigns could potentially correct.

## Methods

Twitter posts containing the term “nicotine” (“#nicotine” would also be included in this search) were obtained from Twitter’s Streaming Application Program Interface (API; the filtered stream using the Twitter4J library for collecting tweets with no gaps in the collection time) from September 30, 2018 to October 1, 2019. There was a total of 1,203,466 posts containing this term during this time. Similar to prior research [15,18], we removed all retweets (n=786,327) and non-English tweets (n=45,497), resulting in 371,642 unique tweets. Removing retweets allowed us to treat each observation as independent. Posts that contained the term “nicotine” but were determined to be unrelated to our research objectives were identified and removed. This included tweets containing the phrases, “bad nicotine,” “nicotine heroin,” “nicotine stain,” and “silver spoon,” as these were references to popular song lyrics. As a result of this filtering process, we were left with 364,430 unique tweets.

Next, we identified social bots [20]. Social bots may bias the data, reducing our ability to dependably describe the public’s recent experience with nicotine [22]. We used Botometer [23] to distinguish between nonbots and social bots. Botometer analyzes the characteristics of a Twitter account and scores it based on how likely the account is to be a social bot. It is considered a state-of-the-art machine learning algorithm and has been used in prior research revolving around social bots and public health [15,21,24]. The Botometer threshold was set to ≥4 on the scale out of 5 of English scores and similar to prior research [25]. Each Twitter account was screened after posts were collected (ie, not in real time). During this process, Twitter accounts (n=27,186) responsible for posts in our data had been deleted. Because these Twitter accounts ceased to exist and could not be processed through Botometer, we removed the posts (n=42,890) from these accounts from our data. The final sample contained 321,540 posts, with 300,360 posts from 181,439 unique nonbot accounts, and 21,180 posts from 5889 social bots.

All analyses relied on public, anonymized data; adhered to the terms and conditions, terms of use, and privacy policies of Twitter; and were performed under the institutional review board approval from the authors’ university. To protect privacy, no tweets were reported verbatim in this article. To promote full transparency and foster reproducibility, all data and code are available from the lead author and posted on his website and data repository.

To prepare tweets for analysis, we conducted a number of transformations, including (1) basic normalization (ie, lower casing all tweets; removing extra spaces, punctuation, and special characters such as brackets), (2) stop word removal (ie, removing words such as “a,” “the”), (3) normalizing Twitter account mentions (ie, @account_name occurrences in the tweets were replaced by @person — a common token for all accounts), (4) lemmatization (ie, the removal of inflections and variants of words), (5) nonprintable character removal (ie, removing emoticons or as symbols from non-English languages), and (6) removal of hashtags and URLs.

To find topics within the tweets, we generated n-grams for n=1 (ie, unigrams) and n=2 (ie, bigrams) from each tweet. An n-gram is simply a sequence of n words. For example, the phrase “Player breaks record” contains the unigrams “player,” “breaks,” “record” and the bigrams “player breaks” and “breaks record.” By generating frequency counts of the most common unigrams and bigrams, we obtained an initial sense of the commonly discussed topics. From this assessment of the most common words and phrases, 4 of the authors reviewed posts in their entirety and arrived at a consensus on 15 commonly occurring topics. This strategy was used to summarize the raw text-based data, documenting the patterns that were present. Topics included person tagging (@person), addiction (mentions of being addicted to nicotine or craving nicotine), appeal (mentions of liking or loving nicotine), nicotine replacement therapies (NRT; mentions of the patch, gum, nicotine replacement), vaping (mentions of using e-cigarettes, vaping, JUUL), smoking (mentions of smoking cigarettes, using other combustible tobacco), nicotine health risks (mentions of nicotine effects on the brain, respiratory health, the amount of nicotine in products), withdrawal (mentions of nicotine withdrawal), quit nicotine (mentions of quitting nicotine or going nicotine free), cessation (mentions of quitting or stopping smoking), polysubstance use (mentions of alcohol and nicotine use), caffeine (mentions of coffee and nicotine use), underage use (mentions of children and teens using nicotine, use of nicotine at high schools), and new products (mentions of a “nicotine shot” or a supplement to boost the amount of nicotine in e-liquids). Nicotine is safe (mentions of nicotine not being harmful by itself) was a topic established a priori since these posts may reflect misconceptions that could be addressed by health education campaigns [26].

Each tweet was classified to one or more topics based on the occurrence of at least one topic-related pattern, which is similar to prior research [18,25]. This pattern could be a unigram, a bigram, or groups of words that must occur in the normalized tweets in a specific order. This was accomplished by using a rule-based classification algorithm developed in Python that inspects each tweet for the presence of a specified set of patterns representing a topic*.* Since a single post could discuss multiple topics, we report the percentage of overlap between each topic by utilizing a confusion matrix. Each cell in the matrix represents the intersection of 2 topics. The value of the cell represents the percentage of the total corpus that belongs to both topics. For example, a hypothetical post such as “Hey @person look who is nicotine free today” would be classified under “person tagging” and “quit nicotine*.*” The number of posts containing both would be found at the intersection of the matrix for these 2 topics.

## Results

**Figure 1:**
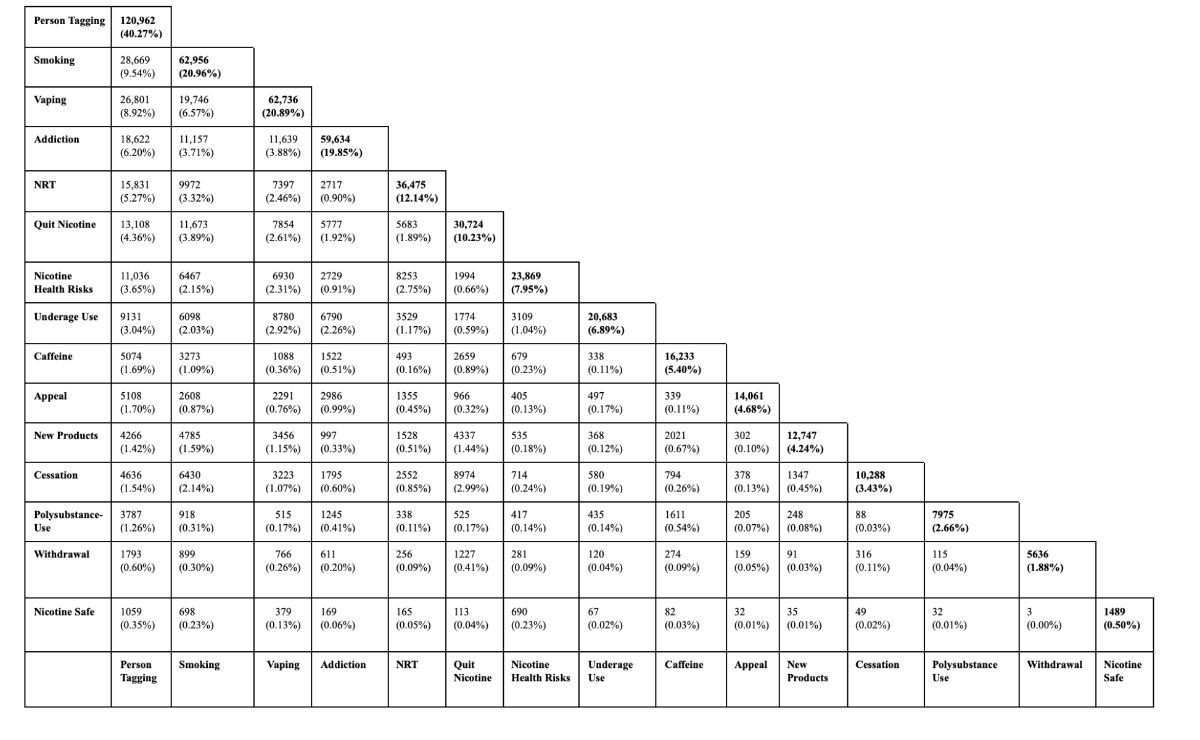
Prevalence of topics from nonbot corpus. NRT: nicotine replacement therapy.

The total coverage of the 15 topics constituted 82.86% (248,893/300,360) of all tweets in the corpus from nonbots (Figure 1). The remaining 17.14% (51,467/300,360) of tweets were too diverse to be classified into a single topic with meaningful coverage (ie, coverage of each subsequent topic would be less than 1% of total tweets). The most prevalent topic in this corpus was “person tagging” at 40.27% (120,962/300,360), followed by “smoking” at 20.96% (62,956/300,360) and “vaping” at 20.89% (62,736/300,360). “Addiction” was the next most prevalent topic at 19.85% (59,634/300,360), followed by “NRT” at 12.14 % (36,475/300,360) and “quit nicotine” at 10.23% (30,724/300,360). Among “quit nicotine,” posts suggested going “cold turkey,” a day without nicotine, trying to quit, and needing help in quitting. “Nicotine health risks” was a common topic at 7.95% (23,869/300,360), followed by “underage use” at 6.89% (20,683/300,360), “caffeine” at 5.40% (16,233/300,360), “appeal” at 4.68% (14,061/300,360), “new products” at 4.24% (12,747/300,360), and “cessation” at 3.43% (10,288/300,360). Among “cessation,” posts suggested quitting and stopping smoking. “Nicotine is safe” was an uncommon topic at 0.50% (1489/300,360).

**Figure 2:**
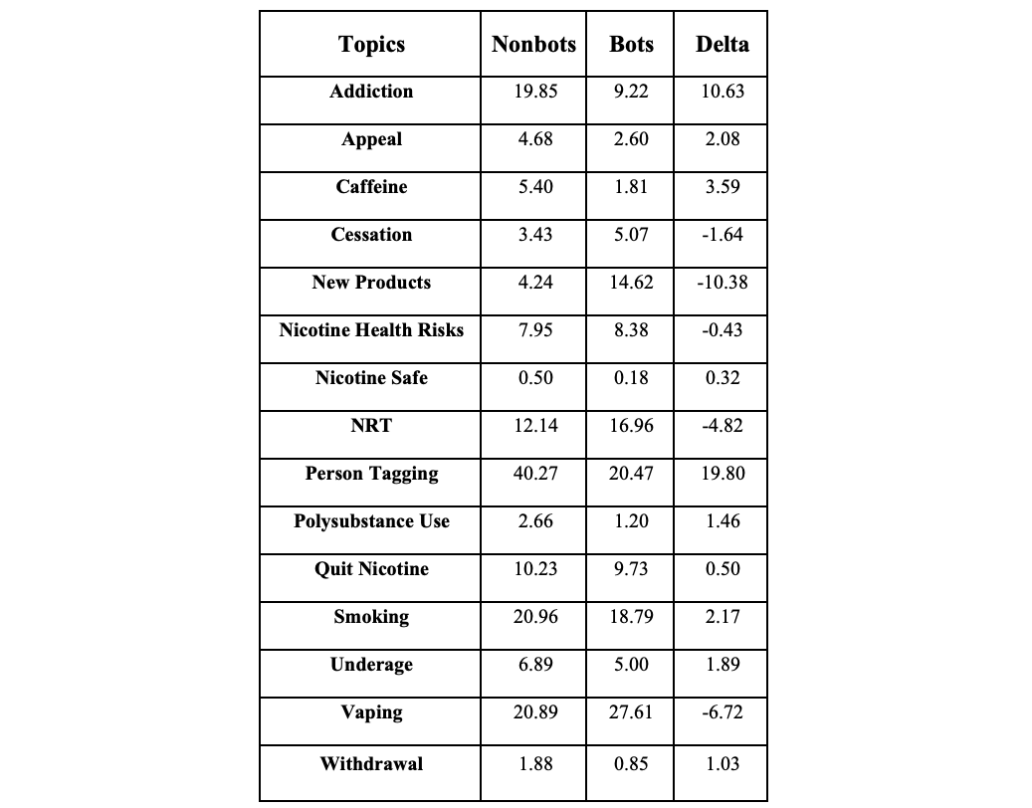
Comparison of prevalence of topics between nonbots and social bots. NRT: nicotine replacement therapy.

The total coverage of the same 15 topics constituted 75.56% (16,004/21,180) of all tweets in the corpus from social bots (Figure 2). Comparing the 2 corpora, some topics had similar prevalences, while other topics stood out with large differences. For example, the largest difference in prevalence in topics between corpora was found in “person tagging” (nonbots at 40.27% [120,962/300,360] versus social bots at 20.47% [4336/21,180]), followed by “new products” (nonbots at 4.24% [12,747/300,360] versus social bots 14.62% [3096/21,180]) and “addiction” (nonbots at 19.85% [59,634/300,360] versus social bots at 9.22% [1952/21,180]). The content found in each category was overall consistent between nonbots and social bots in all but “cessation.” Posts in “cessation” from social bots regularly included the use of hypnotherapy, acupuncture, magnets worn on the ears, and time spent in the sauna as effective ways to stop smoking.

## Discussion

### Principal Findings

This study is one of the largest Twitter studies to date focused on nicotine-related conversations, describing over 300,000 unique posts from over 180,000 unique accounts and addressing the underlying questions of what the public discusses or perceives about nicotine (rather than focusing on one specific tobacco product). We identified a number of topics of conversation ranging from nicotine appeal to withdrawal to smoking cessation. Posts discussed addiction, NRT, health risks, and nicotine use in combination with alcohol and caffeine. This study also distinguished nicotine-related topics of conversations by social bots and nonbots, describing differences in prevalence of topics by account type.

In this study, Twitter posts mentioning new products represented a larger proportion of posts by social bots compared to nonbots, suggesting that companies or retailers or e-cigarette hobbyists may be using bots to promote new products. Social bots have previously been found to promote emerging products on Twitter; for example, in 2017, it was found that social bots were more than 2 times as likely to post about a new vaping product compared to nonbots [15]. Posts from social bots identified in the present study perpetuated a number of methods with very limited evidence as smoking cessation interventions, including hypnotherapy, acupuncture, trips to the sauna, and the use of magnets behind the ear. In contrast to front-line treatments such as tailored behavioral counseling (eg, individual, group, and phone) and medication (eg, varenicline, bupropion, NRT), these alternative methods have little to no empirical evidence to support their efficacy [27,28]. Unsubstantiated health claims perpetuated by social bots may have offline consequences, such as leaving Twitter users with the impression that these methods are good cessation strategies, thus diverting them from more effective approaches.

Unsubstantiated health claims on Twitter from social bots have been documented in prior research; for example, several studies have reported that social bots regularly make claims touting the effectiveness of e-cigarettes in smoking cessation [15,24] and claims propagating misinformation pertaining to vaccinations [21]. Recently, it was reported that social bots were responsible for disseminating unsubstantiated health claims pertaining to cannabis with posts suggesting cannabis could allay health concerns ranging from triple-negative breast cancer to plantar fasciitis [25]. Health education efforts are needed to correct misinformation and ultimately direct individuals who want to quit smoking to evidence-based cessation strategies [27,29]. Misperceptions or myths about cessation could be most persuasively countered with two-sided messages that provide a brief acknowledgement of the misconception, then refute it, and followed by a stronger statement about the more effective intervention [30]. For example, Twitter posts could be circulated that state: “If you feel addicted to cigarettes, you could try quitting cold turkey or with hypnotherapy, but you are more likely to succeed if you work with a Quitline like 1-800-NO BUTTS.”

“Person tagging” was a predominant theme in the current study of nicotine-related posts to Twitter and in line with prior research [18,25]. Person tagging in this context is a social practice where Twitter users directly interact with one another to exchange their attitudes about and experiences with nicotine. Posts classified under “person tagging” regularly used @Person to engage others in discussions about nicotine. These online communications may impact nicotine use; for example, Unger and colleagues [31] demonstrated an empirical link between adolescents’ and young adults’ tobacco-related Twitter activity and their tobacco product use. The current study’s findings are highly relevant to the public health community, as repeated exposure to nicotine-related messaging and reported nicotine use by Twitter connections may influence the social norms of those exposed to the content and lead to imitation of the behaviors [32].

Prior research has shown that a cessation program utilizing Twitter to deliver an intervention for smoking cessation can be successful in helping participants sustain abstinence [33]. The present study did not identify participants looking to quit smoking on Twitter; however, these findings suggest that Twitter may be a place where such participants could be found as people tweet about the difficulty of quitting nicotine. “Vaping,” “addiction,” “quit nicotine,” “withdrawal,” and “cessation” were all topics in the present study. Future interventions could be designed to follow these topics of discussions on Twitter and engage with potential participants about evidence-based cessation methods in near real time when people are contemplating cessation [34].

**“**Polysubstance use” and “caffeine” were identified as topics in the current study. Polysubstance use has been reported in several earlier Twitter-based studies; for example, a prior analysis of hookah-related posts to Twitter from 2017 to 2018 found that many posts described alcohol, marijuana, and other substance use along with hookah [18]. Similar findings were reported in Twitter studies focused on e-cigarettes [15] and cannabis [35]. Past work also raises concerns about the unknown health effects of caffeine in flavored e-liquids [36] and preference of e-liquids with active caffeine ingredients for weight loss [37]. The present findings supplement these previous studies and further awareness of the occurrence of polysubstance use. This is particularly important because alcohol and caffeine can potentiate the reinforcing effects of nicotine [38,39], potentially leading to escalation in use of one or both substances.

Similar to prior Twitter studies focused on JUUL use [40], the current study found posts indicative of underage use of nicotine (ie, mentions of nicotine use at high schools and among teenagers). This finding is concerning because nicotine impairs adolescents’ and young adults’ brain development [2,3,41]. In addition, posts about underage use may normalize e-cigarettes in young viewers, with the potential to increase experimentation and regular use [42].

### Limitations

This study focused on posts to Twitter, and findings may not extend to other social media platforms. The posts in this study were collected within a 12-month period and may not extend to other time periods. Data collection relied on Twitter’s Streaming API, which prevented collection of posts from private accounts. Findings may not generalize to all Twitter users or to the US population. Not all tweets were covered by the established categories, and topics of conversation were not segmented by geographic location, preventing this study from understanding the effect of different state tobacco policies on the public’s experience with nicotine. Prior research has shown that significant geographic biases can occur in the context of conversations over Twitter [43,44]. In some instances, unigrams and bigrams used to define topics may have multiple meanings that were ignored in the current study; for example, the word “school” in nicotine-related posts may not always indicate underage use, as college students or other educational professionals may be discussing nicotine use.

### Conclusions

Common nicotine-related topics on Twitter included smoking, vaping, cessation, withdrawal, and appeal, among others. These results suggest that Twitter users often discuss grappling with quitting smoking, nicotine withdrawal, and nicotine cravings. Such topics of conversation warrant considerations by public health researchers in the future. Twitter may act as a platform to engage with those struggling with nicotine dependence, as well as those initiating use with nicotine-related products, by informing them of the potential for dependence and subsequent health consequences of use. Posts from social bots regularly included the use of hypnotherapy, acupuncture, magnets worn on the ears, and time spent in the sauna as effective ways to stop smoking. Misinformation regarding nicotine has been a component of tobacco industry marketing and has the potential to influence beliefs, perceptions, and use of tobacco; thus, it is important to provide a recent account of what posts discuss on Twitter about nicotine in hopes of correcting misinformation and directing tobacco users to more effective interventions.

## Abbreviations

API: application program interface
NRT: nicotine replacement therapy

## References

[1] Dai H, Leventhal AM (2019). Prevalence of e-Cigarette Use Among Adults in the United States, 2014-2018. JAMA.

[2] England LJ, Bunnell RE, Pechacek TF, Tong VT, McAfee TA (2015). Nicotine and the Developing Human: A Neglected Element in the Electronic Cigarette Debate. Am J Prev Med.

[3] National Center for Chronic Disease Prevention and Health Promotion (US) Office on Smoking and Health (2014). The Health Consequences of Smoking—50 Years of Progress: A Report of the Surgeon General.

[4] Jacobsen LK, Krystal JH, Mencl WE, Westerveld M, Frost SJ, Pugh KR (2005). Effects of smoking and smoking abstinence on cognition in adolescent tobacco smokers. Biol Psychiatry.

[5] Czoli CD, Fong GT, Mays D, Hammond D (2017). How do consumers perceive differences in risk across nicotine products? A review of relative risk perceptions across smokeless tobacco, e-cigarettes, nicotine replacement therapy and combustible cigarettes. Tob Control.

[6] Benowitz NL (2009). Pharmacology of nicotine: addiction, smoking-induced disease, and therapeutics. Annu Rev Pharmacol Toxicol.

[7] Cummings KM, Hyland A, Giovino G, Hastrup J, Bauer J, Bansal M (2004). Are smokers adequately informed about the health risks of smoking and medicinal nicotine?. Nicotine Tob Res.

[8] Fadus MC, Smith TT, Squeglia LM (2019). The rise of e-cigarettes, pod mod devices, and JUUL among youth: Factors influencing use, health implications, and downstream effects. Drug Alcohol Depend.

[9] Maloney S, Breland A, Soule E, Hiler M, Ramôa C, Lipato T, Eissenberg T (2019). Abuse liability assessment of an electronic cigarette in combustible cigarette smokers. Exp Clin Psychopharmacol.

[10] Cobb CO, Lopez AA, Soule EK, Yen M, Rumsey H, Lester Scholtes R, Rudy AK, Lipato T, Guy M, Eissenberg T (2019). Influence of electronic cigarette liquid flavors and nicotine concentration on subjective measures of abuse liability in young adult cigarette smokers. Drug Alcohol Depend.

[11] Barrington-Trimis JL, Kong G, Leventhal AM, Liu F, Mayer M, Cruz TB, Krishnan-Sarin S, McConnell R (2018). E-cigarette Use and Subsequent Smoking Frequency Among Adolescents. Pediatrics.

[12] Chaffee BW, Watkins SL, Glantz SA (2018). Electronic Cigarette Use and Progression From Experimentation to Established Smoking. Pediatrics.

[13] Stanton C, Bansal-Travers M, Johnson A, Sharma E, Katz L, Ambrose B, Silveira M, Day H, Sargent J, Borek N, Compton W, Johnson S, Kimmel H, Kaufman A, Limpert J, Abrams D, Cummings K, Goniewicz M, Tanski S, Travers M, Hyland A, Pearson J (2019). Longitudinal e-Cigarette and Cigarette Use Among US Youth in the PATH Study (2013-2015). J Natl Cancer Inst.

[14] Majmundar A, Kirkpatrick M, Cruz TB, Unger JB, Allem J (2020). Characterising KandyPens-related posts to Instagram: implications for nicotine and cannabis use. Tob Control.

[15] Allem J, Ferrara E, Uppu SP, Cruz TB, Unger JB (2017). E-Cigarette Surveillance With Social Media Data: Social Bots, Emerging Topics, and Trends. JMIR Public Health Surveill.

[16] Perrin A, Anderson M (2019). Share of U.S. adults using social media, including Facebook, is mostly unchanged since 2018. Pew Research Center.

[17] Anderson M, Jiang J (2018). Pew Research Center.

[18] Allem J, Dharmapuri L, Leventhal A, Unger J, Boley Cruz T (2018). Hookah-Related Posts to Twitter From 2017 to 2018: Thematic Analysis. J Med Internet Res.

[19] Majmundar A, Allem J, Cruz TB, Unger JB (2019). Where Do People Vape? Insights from Twitter Data. Int J Environ Res Public Health.

[20] Allem J, Ferrara E (2018). Could Social Bots Pose a Threat to Public Health?. Am J Public Health.

[21] Broniatowski DA, Jamison AM, Qi S, AlKulaib L, Chen T, Benton A, Quinn SC, Dredze M (2018). Weaponized Health Communication: Twitter Bots and Russian Trolls Amplify the Vaccine Debate. Am J Public Health.

[22] Allem J, Ferrara E (2016). The Importance of Debiasing Social Media Data to Better Understand E-Cigarette-Related Attitudes and Behaviors. J Med Internet Res.

[23] Ferrara E, Varol O, Davis C, Menczer F, Flammini A (2016). The rise of social bots. Commun. ACM.

[24] Martinez LS, Hughes S, Walsh-Buhi ER, Tsou M (2018). "Okay, We Get It. You Vape": An Analysis of Geocoded Content, Context, and Sentiment regarding E-Cigarettes on Twitter. J Health Commun.

[25] Allem J, Escobedo P, Dharmapuri L (2020). Cannabis Surveillance With Twitter Data: Emerging Topics and Social Bots. Am J Public Health.

[26] Huang J, Feng B, Weaver SR, Pechacek TF, Slovic P, Eriksen MP (2019). Changing Perceptions of Harm of e-Cigarette vs Cigarette Use Among Adults in 2 US National Surveys From 2012 to 2017. JAMA Netw Open.

[27] 2008 PHS Guideline Update Panel‚ Liaisons‚ and Staff (2008). Treating tobacco use and dependence: 2008 update U.S. Public Health Service Clinical Practice Guideline executive summary. Respir Care.

[28] Barnes J, McRobbie H, Dong CY, Walker N, Hartmann-Boyce J (2019). Hypnotherapy for smoking cessation. Cochrane Database Syst Rev.

[29] (2021). Final Recommendation Statement: Interventions for Tobacco Smoking Cessation in Adults, Including Pregnant Persons. US Preventive Services Task Force.

[30] Allen M (1991). Meta‐analysis comparing the persuasiveness of one‐sided and two‐sided messages. Western Journal of Speech Communication.

[31] Unger JB, Urman R, Cruz TB, Majmundar A, Barrington-Trimis J, Pentz MA, McConnell R (2018). Talking about tobacco on Twitter is associated with tobacco product use. Prev Med.

[32] Fujimoto K, Valente TW (2012). Social network influences on adolescent substance use: disentangling structural equivalence from cohesion. Soc Sci Med.

[33] Pechmann C, Delucchi K, Lakon CM, Prochaska JJ (2017). Randomised controlled trial evaluation of Tweet2Quit: a social network quit-smoking intervention. Tob Control.

[34] Deb A, Majmundar A, Seo S, Matsui A, Tandon R, Yan S, Allem J, Ferrara E (2018). Social Bots for Online Public Health Interventions.

[35] Krauss MJ, Grucza RA, Bierut LJ, Cavazos-Rehg PA (2016). “Get drunk. Smoke weed. Have fun.”: A Content Analysis of Tweets About Marijuana and Alcohol. Am J Health Promot.

[36] Lisko JG, Lee GE, Kimbrell JB, Rybak ME, Valentin-Blasini L, Watson CH (2017). Caffeine Concentrations in Coffee, Tea, Chocolate, and Energy Drink Flavored E-liquids. Nicotine Tob Res.

[37] Morean ME, Wedel AV (2017). Vaping to lose weight: Predictors of adult e-cigarette use for weight loss or control. Addict Behav.

[38] Jones HE, Griffiths RR (2003). Oral caffeine maintenance potentiates the reinforcing and stimulant subjective effects of intravenous nicotine in cigarette smokers. Psychopharmacology (Berl).

[39] Rose JE, Brauer LH, Behm FM, Cramblett M, Calkins K, Lawhon D (2002). Potentiation of Nicotine Reward by Alcohol. Alcoholism Clin Exp Res.

[40] Allem J, Dharmapuri L, Unger JB, Cruz TB (2018). Characterizing JUUL-related posts on Twitter. Drug Alcohol Depend.

[41] Jasinska AJ, Zorick T, Brody AL, Stein EA (2014). Dual role of nicotine in addiction and cognition: a review of neuroimaging studies in humans. Neuropharmacology.

[42] Gorukanti A, Delucchi K, Ling P, Fisher-Travis R, Halpern-Felsher B (2017). Adolescents' attitudes towards e-cigarette ingredients, safety, addictive properties, social norms, and regulation. Prev Med.

[43] Gore RJ, Diallo S, Padilla J (2015). You Are What You Tweet: Connecting the Geographic Variation in America's Obesity Rate to Twitter Content. PLoS One.

[44] Padilla JJ, Kavak H, Lynch CJ, Gore RJ, Diallo SY (2018). Temporal and spatiotemporal investigation of tourist attraction visit sentiment on Twitter. PLoS One.

